# Development and Validation of a Sulfa Antibiotic Allergy Clinical Decision Rule

**DOI:** 10.1001/jamanetworkopen.2023.16776

**Published:** 2023-06-05

**Authors:** Jamie L. Waldron, Morgan Rose, Sara Vogrin, Matthew S. Krantz, Ryan Bolotte, Elizabeth J. Phillips, Jason A. Trubiano

**Affiliations:** 1 Centre for Antibiotic Allergy and Research, Department of Infectious Diseases, Austin Health, Heidelberg, Victoria, Australia; 2 Department of Medicine (St Vincent’s Health), University of Melbourne, Fitzroy, Victoria, Australia; 3 Division of Allergy, Pulmonary and Critical Care Medicine, Department of Medicine, Vanderbilt University Medical Center, Nashville, Tennessee; 4 Center for Drug Safety and Immunology, Vanderbilt University Medical Center, Nashville, Tennessee; 5 Department of Pediatrics, Vanderbilt University Medical Center, Nashville, Tennessee; 6 Department of Infectious Diseases, University of Melbourne, Parkville, Victoria, Australia

## Abstract

This cohort study describes the adaptation of a widely used penicillin allergy clinical decision tool for evaluation of trimethoprim-sulfamethoxazole allergy.

## Introduction

Trimethoprim-sulfamethoxazole is first-line treatment for many infections; however, use is limited by sulfa allergy.^1^ Use of trimethoprim-sulfamethoxazole is frequently required by antimicrobial stewardship programs to prevent use of more restricted antibotics.^2^ Studies with nonstandardized challenge criteria suggest that those with low-risk allergy phenotypes can safely undergo direct oral challenges (OCs); however, no current risk-stratification tool exists to guide challenges.^3,4^ We sought to adapt PEN-FAST, a penicillin allergy clinical decision tool, for trimethoprim-sulfamethoxazole allergy.^5^

## Methods

PEN-FAST (eFigure in Supplement 1)^5^ was adapted as a trimethoprim-sulfamethoxazole allergy clinical decision rule (SULF-FAST) for use and validation in 2 data sets (in Australia and the US). Patients aged 18 years or older with a trimethoprim-sulfamethoxazole allergy referred to drug allergy services in Melbourne, Australia (Austin Health, Peter MacCallum Cancer Centre; November 1, 2015, to July 31, 2022), or Nashville, Tennessee (Vanderbilt University Medical Center; October 1, 2015, to February 28, 2019), were prospectively assessed.^4,5^ Patients with a nonsevere sulfa or trimethoprim-sulfamethoxazole allergy (ie, excluding anaphylaxis within 5 years and severe cutaneous adverse drug reaction),^4^ provided written consent to undergo OC at clinician discretion (eTable in Supplement 1). A positive test result was defined as a positive patch test (PT) result or a clinician-observed or patient-reported presumed immune-mediated reaction after the challenge. A PEN-FAST score (eFigure in Supplement 1) and its diagnostic performance were calculated for each cohort and allergy phenotype subgroup. Statistical analysis is detailed in the eMethods in Supplement 1. This study was approved by the Vanderbilt University Medical Center institutional review board and the Austin Health human research ethics committees. This study followed the STROBE reporting guideline.^6^

## Results

In this study, the Australian (n = 116) and US (n = 204) cohorts had a similar age distribution (median age, 64 years [IQR, 54-74 years] and 62 years [IQR, 48-70 years], respectively) (Table 1). Prevalence of a positive trimethoprim-sulfamethoxazole allergy test result was 5.2% (95% CI, 1.9%-10.9% [6 of 116]) in Australia and 6.4% (95% CI, 3.4%-10.7% [13 of 204]) in the US.

**Table 1.** Patient Demographic and Testing Characteristics of Trimethoprim-Sulfamethoxazole Allergy in Tested Derivation and Validation Cohorts^a^

Characteristic	**Melbourne, Australia** ^b^			**Nashville, Tennessee** ^c^		
	Overall (N = 116)	Positive (n = 6)	Negative (n = 110)	Overall (N = 204)	Positive (n = 13)	Negative (n = 191)
Age, median (IQR), y	64 (54-74)	54 (46-66)	64 (54-75)	62 (48-70)	48 (13-61)	62 (50-70)
Sex, No. (%)						
Female	72 (62.1)	2 (33.3)	70 (63.6)	162 (79.4)	11 (84.6)	151 (79.1)
Male	44 (37.9)	4 (66.7)	40 (36.4)	42 (20.6)	2 (15.4)	40 (20.9)
Race and ethnicity, No. (%)						
Asian	6 (5)	NA	NA	2 (1)	NA	NA
Black	1 (1)			9 (4)		
White	104 (90)			188 (92)		
Unknown	5 (4)			5 (2)		
Comorbidities, No. (%)						
HIV	0	NA	NA	9 (4.4)	NA	NA
Diabetes	5 (4.3)			38 (18.6)		
Transplant	18 (15.5)			17 (8.3)		
Immunocompromised (other than HIV or transplant)	59 (50.9)			NA		
PEN-FAST components, No. (%)						
Treatment required	9 (7.8)	5 (83.3)	4 (3.6)	21 (10.3)	5 (38.5)	16 (8.4)
Anaphylaxis, angioedema, or SCAR	7 (6.0)	2 (33.3)	5 (4.5)	40 (19.6)	4 (30.8)	36 (18.8)
Reaction within last 5 y	57 (49.1)	5 (83.3)	52 (47.2)	69 (33.8)	8 (61.5)	61 (31.9)
Allergy phenotype, No. (%)						
Immediate	32 (27.6)	0	32 (29.1)	23 (11.3)	3 (23.1)	20 (10.5)
Delayed	41 (35.3)	6 (100)	35 (31.8)	106 (52)	9 (69.2)	97 (50.8)
Unknown or other^d^	43 (37.1)	0	43 (39.1)	75 (36.8)	1 (7.7)	74 (38.7)
Allergy phenotype details, No. (%)						
Childhood	19 (16.4)	NA	NA	Unavailable		
Diffuse rash	57 (49.1)					
Urticaria	6 (5.2)					
Unknown	18 (15.5)					
Blistering or desquamation	4 (3.4)					
Swelling	4 (3.4)					
Respiratory compromise	2 (1.7)					
Fever	3 (2.6)					
Anaphylaxis	1 (0.9)					
Testing method, No. (%)						
PT	16 (13.8)	6 (100)	10 (9.1)	0	0	0
OC	111 (95.7)	1 (16.7)	110 (100)	204 (100)	13 (100)	191 (100)
Positive PT result		5/6			0	
Positive OC result^e^		1/6			13/13	
Immediate reaction to OC	NA	0	NA	NA	7/13	NA
Pruritus					2/7	
Urticaria					2/7	
Arm pain and malaise					1/7	
Fever					1/7	
Throat pruritus with chest tightness					1/7	
Delayed reaction to OC	NA	1/1	NA	NA	6/13	NA
Benign exanthem		1/1			1/6	
Erythema					2/6	
Urticaria					1/6	
Fever with myalgias and headache					1/6	
Fever with nausea and vomiting					1/6	

Abbreviations: NA, not applicable; OC, oral challenge; PT, patch test; SCAR, severe cutaneous adverse reaction.

^a^ Includes specific antibiotic allergy label of Co-T or trimethoprim-sulfamethoxazole. Recent anaphylaxis was defined as anaphylaxis within the past 5 years; a positive allergy test result was defined as any positive allergy test finding, including positive result on patch testing or oral challenge.

^b^ Prospective cohort; Austin Health (87.1% [101 of 116]) and Peter MacCallum Cancer Centre (12.9% [15 of 116]); November 1, 2015, to July 31, 2022. Testing sites were inpatient and outpatient. Excluded phenotypes were recent anaphylaxis and SCAR. Patients underwent patch testing and oral challenge testing after patch testing.

^c^ Retrospective cohort; Vanderbilt University Medical Center; October 1, 2015, to February 28, 2019. Testing sites were outpatient only. Excluded phenotypes were recent anaphylaxis and SCAR. Patients underwent 1-step and 2-step oral challenge testing.

^d^ Includes nonimmune mediated and reactions with unknown timeline.

^e^ Most patients with positive test reported a “trimethoprim-sulfamethoxazole” allergy (6 of 6 in the Melborne cohort and 12 of 13 in the US cohort), while 1 patient reported “unspecified sulfa” allergy.

The PEN-FAST tool that applied to the Australian cohort showed good discrimination in determining true allergy, with an area under the curve (AUC) of 0.86. A low score (<3) carried low allergy risk (<5%), while a score of 3 or more had high allergy risk (>20%). A cutoff of less than 3 points classified 108 patients as low risk, with 2 having a positive result for 1 or more allergy tests (1 morbilliform drug eruption after OC, 1 positive PT). Sensitivity to identifying this allergy using this cutoff was 66.7% (95% CI, 22.3%-95.7%), with a specificity of 96.4% (95% CI, 91.0%-99.9%), positive predictive value (PPV) of 50.0% (95% CI, 15.7%-84.3%), and negative predictive value (NPV) of 98.1% (95% CI, 93.5%- 99.8%) (Table 2). Subgroup analysis showed good performance with delayed phenotype (AUC, 0.78 [95% CI, 0.56-0.99]); however, limited cohort numbers prevented performance assessment for immediate and unknown phenotypes.

**Table 2.** Validation of SULF-FAST in All Validation Cohorts (Overall) With Subgroup Analysis Performed on Allergy Phenotype^a^

Characteristic	Melbourne, Australia				Nashville, Tennessee			
	Overall (N = 116)	Immediate (n = 32)	Delayed (n = 41)	Unknown (n = 43)	Overall (N = 204)	Immediate (n = 23)	Delayed (n = 106)	Unknown (n = 75)
No. (%) with SULF-FAST score ≥3	8 (6.9)	0	8 (19.5)	0	25 (12.3)	7 (30.5)	14 (13.2)	4 (5.3)
No./total No. (%) with SULF-FAST score ≥3 with positive test result	4/8 (50)	NA	4/8 (50)	NA	5/25 (20)	2/7 (28.6)	3/14 (21.4)	0/4 (0)
Validation AUC (95% CI)	0.82 (0.61-1.00)	NA	0.78 (0.56-0.99)	NA	0.64 (0.5-0.78)	0.71 (0.37-1.00)	0.61 (0.44-0.78)	0.47 (n/a)
Sensitivity, % (95% CI)	66.7 (22.3-95.7)	NA	66.7 (22.3-95.7)	NA	38.5 (13.9-68.4)	66.7 (9.4-99.2)	33.3 (7.5-70.1)	0 (0-97.5)
Specificity, % (95% CI)	96.4 (91.0-99.0)	NA	88.6 (73.3-96.8)	NA	89.5 (84.3-93.5)	75 (50.9-91.3)	88.7 (80.6-94.2)	94.6 (86.7-98.5)
PPV, % (95% CI)	50.0 (15.7-84.3)	NA	50 (15.7-84.3)	NA	20.0 (6.8-40.7)	28.6 (3.7-71.0)	21.4 (4.7-50.8)	0 (0-60.2)
NPV, % (95% CI)	98.1 (93.5-99.8)	NA	93.9 (79.8-99.3)	NA	95.5 (91.4-98.1)	93.8 (69.8-99.8)	93.5 (86.3-97.6)	98.6 (92.4-100)

Abbreviations: AUC, area under the curve for S-FAST score 3 or higher; NA, not applicable; NPV, negative predictive value; PPV, positive predictive value.

^a^ Immediate phenotype was defined as any reaction that occurred within 1 hour after exposure to initial medication dose, whereas delayed phenotype was defined as any reaction occurring beyond 1 hour, including those after multiple medication doses. Unknown phenotype includes any reaction that was nonimmune mediated and reactions with unknown timeline.

The AUC for the US cohort was 0.67, with 179 patients scored as low risk, with 8 (4.5%) testing positive for trimethoprim-sulfamethoxazole allergy (all during direct OC; Table 1). A cutoff of less than 3 points showed sensitivity of 38.5% (95% CI, 13.9%-68.4%), specificity of 89.5% (95% CI, 84.3%-93.5%), PPV of 20.0% (95% CI, 6.8%-40.7%), and NPV of 95.5% (95% CI, 91.4%-98.1%) (Table 2). In subgroup analysis, performance for immediate reaction was good (AUC, 0.71 [95% CI, 0.37-1.00]), with lower performance in delayed phenotype (AUC, 0.61 [95% CI, 0.44-0.78]). Of 115 patients with 3- to 72-month prescribing data, 45 (39.1%) were administered a trimethoprim-sulfamethoxazole course (>1 dose), with no adverse reactions.

## Discussion

Application of PEN-FAST criteria provides a good initial framework for identifying appropriate challenge candidates among patients with low-risk trimethoprim-sulfamethoxazole allergy. Although sensitivities of 66.7% and 38.5% for the challenge reaction were lower than with PEN-FAST (70.7% [95% CI, 57.3%-81.9%]),^4^ the tool’s safety is reinforced by high specificity and NPV within both cohorts. Although our study was limited by the small cohort and single-dose challenge, SULF-FAST has potential utility for identifying individuals at low risk with low pretest probability of true allergy who could proceed to OC as a delabeling strategy, especially among high-yield populations, such as those undergoing a planned transplant or immunocompromised hosts for whom trimethoprim-sulfamethoxazole is first-line treatment. Although single-dose challenge was protocolized, the high rate of tolerance of multiple extended trimethoprim-sulfamethoxazole treatment doses is reassuring for excluding delayed hypersensitivity. Further validation is required for widespread implementation.

## Notes

Supplement 1.
eMethods.
**eFigure.** PEN-FAST Penicillin Allergy Clinical Decision Rule, Adapted From “Development and Validation of a Penicillin Allergy Clinical Decision Rule” by Trubiano, et al **eTable.** Criteria for Challenge Type and Corresponding Doses in Both Australia and USA Cohorts. Adapted From Krantz, et al

Supplement 2.
Data Sharing Statement
