# Supplementary material for: Development and Validation of a Sulfa Antibiotic Allergy Clinical Decision Rule
Source: JAMA Netw Open. 2023 Jun 5;6(6):e2316776. doi: 10.1001/jamanetworkopen.2023.16776 (PMC10242418; doi:10.1001/jamanetworkopen.2023.16776)
Supplement: Supplement 1. — eMethods. eFigure. PEN-FAST Penicillin Allergy Clinical Decision Rule, Adapted From “Development and Validation of a Penicillin Allergy Clinical Decision Rule” by Trubiano, et al eTable. Criteria for Challenge Type and Corresponding Doses in Both Australia and USA Cohorts. Adapted From Krantz, et al [file jamanetwopen-e2316776-s001.pdf]

Supplementary Online Content

Waldron JL, Rose M, Vogrin S, et al. Development and validation of a sulfa antibiotic allergy clinical decision rule. *JAMA Netw Open.* 2023;6(6):e2316776. doi:10.1001/jamanetworkopen.2023.16776

eMethods.
eFigure. PEN-FAST Penicillin Allergy Clinical Decision Rule, Adapted From "Development and Validation of a Penicillin Allergy Clinical Decision Rule" by Trubiano, et al
eTable. Criteria for Challenge Type and Corresponding Doses in Both Australia and USA Cohorts. Adapted From Krantz, et al

This supplementary material has been provided by the authors to give readers additional information about their work.

© 2023 Waldron JL et al. *JAMA Network Open.*

eMethods

Statistical analysis

All available data was used for analysis, no prior sample size calculations were performed. Univariable and multivariable logistic regression was performed using each PEN-FAST component. Total score using PEN-FAST scoring algorithm (eFigure I) was calculated and diagnostic performance is presented for each cohort (sensitivity, specificity, positive and negative predictive value, AUC). Additional analysis included stratification by allergy phenotype. Results are reported in accordance to STROBE guidelines.[6] This study was approved by the institutional review board and the human research ethics committees at Vanderbilt University Medical Center (161455) and Austin Health (HREC/15/AUSTIN/75 & HREC/47585/Austin-2018), respectively.

© 2023 Waldron JL et al. *JAMA Network Open.*

eFigure. PEN-FAST Penicillin Allergy Clinical Decision Rule, Adapted From "Development and Validation of a Penicillin Allergy Clinical Decision Rule" by Trubiano, *et al*

**PEN**	*Penicillin allergy reported by patient*	*If yes, proceed with assessment*
**F**	*Five years or less since reaction*[a]	**2 points**
**A**	*Anaphylaxis or angioedema*	
	OR	**2 points**
**S**	*Severe cutaneous adverse reaction*[b]	
**T**	*Treatment required for reaction*[a]	**1 point**
		**Total points**

Interpretation

Points	
0	**Very low risk** of positive penicillin allergy test <1% (<1 in 100 patients reporting penicillin allergy)
1-2	**Low risk** of positive penicillin allergy test 5% (1 in 20 patients)
3	**Moderate risk** of positive penicillin allergy test 20% (1 in 5 patients)
4-5	**High risk** of positive penicillin allergy test 50% (1 in 2 patients)

The PEN-FAST clinical decision rule for patients reporting a penicillin allergy uses 3 clinical criteria of time from penicillin allergy episode, phenotype, and treatment required. A total score is calculated using PEN-FAST score in the upper panel, and interpretation for risk strategy is provided in the lower panel.

[a] Includes unknown.

[b] Forms of severe delayed reactions include potential Stevens-Johnson syndrome, toxic epidermal necrolysis, drug reaction with eosinophilia and systemic symptoms, and acute generalized exanthematous pustulosis. Patients with a severe delayed rash with mucosal involvement should be considered to have a severe cutaneous adverse reaction. Acute interstitial nephritis, drug induced

© 2023 Waldron JL et al. *JAMA Network Open.*

liver injury, serum sickness and isolated drug fever were excluded phenotypes from the derivation and validation cohorts.

© 2023 Waldron JL et al. *JAMA Network Open.*

eTable. Criteria for Challenge Type and Corresponding Doses in Both Australia and USA Cohorts.

Adapted From Krantz, *et al*

**Challenge Type**	**Criteria**	**Dose(s)**	**Obsesrvation Protocol***
Single-dose challenge	Nonsevere delayed reactions without multiple features consistent with IgE-mediated reaction<br>Nonsevere immediate (eg, isolated urticaria, maculopapular rash, or gastrointestinal symptoms) reaction (<1 hr) more than 5 years ago<br>Unknow, remote history	TMP-SMX (Co-T) 80-400mg	1-2 hr observation in clinic after full dose
2-dose challenge	Nonsevere immediate reaction (<1 h) within past 5 years<br>Nonsevere accelerated reaction (>1 but <36 h) within the past 5 y<br>Anaphylaxis at any time point in the past<br>Multiple (2 or more) features potentially compatible with IgE-mediated reaction at any time point in the past<br>• Urticaria<br>• Angioedema<br>• Shortness of breath<br>• Hypotension<br>• Significant patient anxiety surrounding single-dose challenge	TMP-SMX (Co-T) 8-40 mg<br>TMP- SMX (Co-T) 80-400 mg	30 min to 1 hr observation in clinic after first dose<br>1 hr to 2 hr observation in clinic after second, full dose
Prolonged challenge	Nonsevere delayed reaction (>36 h) at any time point in past	TMP-SMX (Co-T) 80-400mg<br>TMP-SMX (Co-T) 160-800 mg daily for 3 days	1 hr observation for single dose challenge in clinic, follow up call 24-48 hours after completion of 3 day outpatient course

*Routine observation protocol varies based on individual clinic location

© 2023 Waldron JL et al. *JAMA Network Open.*
