# Supplementary material for: Development and Validation of a Sulfa Antibiotic Allergy Clinical Decision Rule
Source: JAMA Netw Open. 2023 Jun 5;6(6):e2316776. doi: 10.1001/jamanetworkopen.2023.16776 (PMC10242418; doi:10.1001/jamanetworkopen.2023.16776)
Supplement: Supplement 2. — Data Sharing Statement [file jamanetwopen-e2316776-s002.pdf]

Data Sharing Statement

Waldron. Development and Validation of a Sulfa Antibiotic Allergy Clinical Decision Rule. *JAMA Netw Open*. Published June 05, 2023. doi:10.1001/jamanetworkopen.2023.16776

Data
Data available: No
